# Does a Standardized Discharge Communication Tool Improve Resident Performance and Overall Patient Satisfaction?

**DOI:** 10.5811/westjem.2020.9.48604

**Published:** 2020-11-20

**Authors:** Michael T. Dalley, Mauricio J. Baca, Chandelle Raza, Laurie Boge, David Edwards, Robert Goldszer, Luigi Cubeddu, David Farcy

**Affiliations:** Mount Sinai Medical Center, Department of Emergency Medicine, Miami Beach, Florida

## Abstract

**Introduction:**

The discharge conversation is a critical component of the emergency department encounter. Studies suggest that emergency medicine (EM) residency education is deficient in formally training residents on the patient discharge conversation. Our goal was to assess the proficiency of EM residents in addressing essential elements of a comprehensive discharge conversation; identify which components of the discharge conversation are omitted; introduce “DC HOME,” a standardized discharge mnemonic; and determine whether its implementation improved resident performance and patient satisfaction.

**Methods:**

This was a prospective observational pre- and post-intervention study done by convenience sampling of 400 resident discharge encounters. Resident physicians were observed by attending physicians who completed an evaluation, answering “yes” or “no” as to whether residents addressed six components of a comprehensive discharge. The six components include the following: diagnosis; care rendered; health and lifestyle modifications; obstacles after discharge; medications; and expectations – or “DC HOME.” Didactics introducing the mnemonic “DC HOME” was provided to resident physicians. Patient feedback and satisfaction were collected after each encounter, and we recorded differences between pre-intervention and post-intervention encounters.

**Results:**

Resident physicians improved significantly in all six components of “DC HOME” from pre-and-post intervention: discharge diagnosis (P = 0.0036) and the remaining five components (P<0.0001). There was a statistically significant improvement in patients’ perception for health and lifestyle modifications, obstacles after discharge, medications, expectations after discharge (P<0.0001), and discharge diagnosis (P = 0.0029). Patient satisfaction scores improved significantly (P = 0.005). Time spent with patients during discharge increased from 2 minutes and 42 seconds to 4 minutes and 4 seconds (P<0.0001).

**Conclusion:**

EM residents frequently omit key components of the discharge conversation. The implementation of the “DC HOME” discharge mnemonic improves resident discharge performance, patient perception, and overall patient satisfaction.

## INTRODUCTION

The discharge conversation is a critical component of the emergency department (ED) encounter. Risks of not performing a comprehensive discharge include recidivism, medical errors, adverse drug events, and malpractice liability.^1^–^4^ At discharge, patients and their caregivers have an important role in ensuring a successful transition of care. Assumption of this responsibility has proven to be a source of anxiety for patients and their caregivers, whether from lack of understanding and preparation for the self-care role, confusion due to conflicting practitioner advice, a sense of abandonment, and/or a feeling of overall disregard for their preferences and input.^5^,^6^ Quality discharge instructions have proven to maximize the likelihood patients will fill their prescriptions, be compliant with medications, improve compliance with ED recommendations, and avoid unnecessary adverse complications.^7^–^9^

Despite this, a standardized method to consistently perform a comprehensive and effective patient-centered discharge from the ED is lacking. Vashi et al suggest integral components of the discharge conversation should include explanation of symptoms and expected course of illness, instructions about medications and self-care, and instructions about symptoms that should prompt return to the ED.^10^ Rhodes et al similarly suggested diagnosis, expected course of illness, self-care, use of medications, time-specified follow-up, and reasons to return to the ED as important aspects of the discharge conversation, and they found that in nearly 45% of discharges, emergency medicine (EM) residents were remiss in relaying this critical information.^11^

EM residency education is deficient in formally training and assessing residents on the patient discharge discussion. A 2015 survey of 119 Accreditation Council for Graduate Medical Education (ACGME)-accredited EM programs conducted by the Council of Emergency Medicine Residency Directors (CORD) found that 73.9% of EM programs do not evaluate residents on competency in performing effective discharges.^13^ It also noted that while only 42.9% of programs provided formal instruction on the discharge process during orientation, a mere 5.9% of programs had structured training beyond this initial orientation period.^13^

In this study, we introduce “DC HOME,” which stands for patient’s discharge diagnosis; the care rendered including test results; health and lifestyle modifications; obstacles after discharge; information regarding prescribed medications; and expectations regarding patient diagnosis with follow-up plans after discharge. We hypothesized that formalized education introducing and implementing the use of the “DC HOME” mnemonic during the discharge conversation would consistently address essential elements of aftercare responsibility and would ameliorate the frequency of patients being discharged with inadequate instructions.^11^,^14^

## METHODS

The aims of this study were four-fold: 1) to assess the proficiency of EM residents in addressing essential elements of a comprehensive discharge plan during the discharge conversation; 2) to identify which components of the discharge conversation are repeatedly omitted; 3) to introduce “DC HOME,” a protocolized discharge mnemonic, into EM resident education; and 4) to determine whether its implementation would improve resident performance and patient satisfaction.

**Population Health Research Capsule**

What do we already know about this issue?

The discharge conversation is a critical component of the emergency department (ED) encounter. Emergency medicine residency education is deficient in formally training and assessing residents on the patient dicharge conversation.

What was the research question?

Can formal education and the use of “DC HOME”, a standardized discharge mnemonic, improve resident performance and patient satisfaction during the discharge conversation?

What was the major finding of the study?

The implementation of the “DC HOME” discharge mnemonic improves resident discharge performance, patient perception, and overall patient satisfaction.

How does this improve population health?

Quality discharge instructions have proven to maximize the likelihood patients will fill their perscriptions, be compliant with mediciations, improve compliance with ED recommendations, and avoid unnecessary adverse complications.

This prospective observational before-and-after study was conducted at a Level III trauma, urban, academic ED with a 60,000-annual visit (30% admission rate) in Miami Beach, Florida. The EM residency program is a three-year, ACGME-accredited program with seven resident physicians per year. Prior to this study, the study site used an electronic health record (Epic, Verona, WI) for note documentation, and provided diagnosis-specific, printed discharge instructions to each patient with instructions provided by an ED provider (emergency physician, advanced practice provider, or nurse) involved in the care of the patient. A convenience sample of 400 resident discharge conversations were observed by our EM residency clinical faculty (18 total, four of whom are authors of this study), 200 of which were completed pre-intervention and 200 post-intervention. We collected data from November 2018–June 2019 of the academic year, observing the same cohort of 21 residents. Pre-intervention observations took place from November 2018–January 2019. Post-intervention observations took place one week after a 30-minute didactic session from mid-February 2019–June 2019. Inclusion criteria included adult patients who were being discharged from the ED. Exclusion criteria included patients with altered mental status, less than 18 years of age, non-English speaking, and individuals who refused to participate. Observations took place any time within a 24-hour day/7 days per week schedule.

Prior to the pre-intervention phase of the study, the authors performed a thorough literature search^10^–^13^ identifying essential components of a discharge conversation and incorporated this data with survey question outcomes data from the hospital site’s ED patient satisfaction survey provider (National Research Corporation Picker Survey, Lincoln, NE). Themes ascribed from this information led to a contributing authors’ consensus, which identified six essential components that should be addressed in a comprehensive discharge conversation (see Appendix 1). Each component represents a letter in the “DC HOME” mnemonic created by the authors of this study and introduced in the intervention didactic session.

In the pre-intervention phase of the study, clinical faculty emergency physicians were instructed to observe the discharge encounter between resident physician and patient. A binary questionnaire was provided and the faculty were instructed to answer “yes” or “no” if a resident discussed each of the study-defined six components of the comprehensive discharge conversation. Faculty were provided with specific examples within the body of the survey questionnaire to provide a scoring reference for successful completion (see Appendix I). Satisfactory fulfillment of each criterion was based on the investigating attending physician’s subjective opinion and experience along with the application of the predefined examples of what constitutes a successful acknowledgement of a specific component. If a defined component of the questionnaire was not applicable (ie, a diagnosis that did not require medications at discharge), faculty were instructed to score “yes” if the resident mentioned the component (ie, “no new medication is required today”) or “no” if the component was not brought up in the discharge conversation at all.

The start and end time of the resident physician discharge conversation was recorded. When the resident physician was done discharging the patient, the clinical faculty member would stay behind in the room and ask the patient the same six questions to evaluate patient perception of the discharge conversation. Lastly, patients were asked about their overall satisfaction with the discharge conversation.

After the pre-intervention phase of the study was completed, a 30-minute lecture consisting of a PowerPoint (Microsoft Corp., Redmond, WA) presentation with background information regarding what constitutes a comprehensive discharge^10^–^13^ and introduction of “DC HOME” incorporating the six components of the comprehensive discharge plan was presented at the EM weekly didactic conference where all resident physicians were present. The lecture was followed by a practical, in which each resident present was paired with a co-resident. Each were given three mock-patient encounters with a discharge diagnosis and asked to perform mock discharge conversations on each other, first using their current usual practice and then repeated using “DC HOME.” These mock discharges were observed by the principal investigators present at conference, and feedback regarding areas of improvement in their usual practice and the impact of using the discharge tool was shared with each resident. Residents were instructed to start using “DC HOME” with all future ED discharges. The didactic session made no mention that this intervention was part of an ongoing study. Residents were informed that they would be observed performing discharge conversations after the lecture.

Post-intervention, clinical faculty observed the discharge conversation between the resident physician and patient and completed the same questionnaire as the pre-intervention phase of the study. Clinical faculty, with the exception of the study authors, were unaware that an education session introducing “DC HOME” had taken place prior to the post-intervention phase. The start and end time of the resident physician discharge conversation was recorded. Again, the patients were then asked the six questions alone by the clinical faculty to evaluate patient perception. Clinical faculty then asked patients about satisfaction of the discharge conversation.

Results of “DC HOME” were shown as number of observations of “yes” or “no” with mean percentages for each of the six discharge components. We calculated differences between pre-intervention and post-intervention using Fisher’s exact test. *P*-values equal to or less than 0.05 were considered significant. Patient satisfaction was resulted as “not satisfied,” “somewhat satisfied,” and “very satisfied.” Differences were calculated by using Fisher’s 2 × 3 variant test. Time spent on the discharge process was recorded with each observation and pre- and post-intervention were compared using the unpaired t-test since individual residents were not recorded for each discharge encounter. The study was powered (Type II error 0.2) to detect an effect size of 28.1% with a type I error of 0.05. The study was approved by the hospital’s institutional review board.

## RESULTS

Pre-intervention, resident physicians were observed to have a total of 784 “yes” responses and 416 “no” responses for all six “DC HOME” criteria. Resident physicians during the pre-intervention period discussed diagnosis 95.5% of the time, care rendered 88.5% of the time, medications 80.5% of the time, and expectations 81% of the time. The categories residents mostly omitted were health and lifestyle changes and obstacles after discharge, with health and lifestyle changes 24% of the time and obstacles after discharge 22.5% of the time. In the post-intervention period, resident physicians were observed to have a total of 1193 “yes” responses and 7 “no” responses; thus, significant improvement was found (*P*<0.0001). All six individual components of the discharge instructions showed statistically significant improvement from pre-intervention to post-intervention (refer to Table 1).

Patient perception of the resident discharge conversation at pre-intervention had a total of 921 “yes” responses and 279 “no” responses for all six “DC HOME” criteria. Patients understood diagnosis 94.4% of the time, care rendered 99% of the time, health and lifestyle changes 53.8% of the time, obstacles after discharge 47.2% of the time, medications 78.4% of the time, and expectations 86.9% of the time. The weakest categories were health and lifestyle changes, obstacles after discharge, and medications. Post-intervention for the 6 “DC HOME” components there were a total of 1139 “yes” responses and 61 “no” responses; thus, significant improvement was found (p<0.00001). Patient perception showed statistically significant improvements in all six individual components except care rendered, which showed only slight improvement from 99% to 99.5% (*P* = 0.6231) (refer to Table 2).

Patient satisfaction improved from pre-intervention to post-intervention: 85% of patients were “very satisfied” pre-intervention, and 98% of patients’ post-intervention who received “DC HOME” instructions were “very satisfied” (Table 3).

The average amount of time spent with patients on discharge instructions was 2 minutes and 42 seconds in the pre-intervention group and 4 minutes and 4 seconds in the post-intervention group. This represented a 66% increase in time spent on discharge communication and was statistically significant (*P*<0.0001) (Table 4).

## DISCUSSION

The results of this study emphasize that residents underperform in addressing key elements of the discharge conversation. Implementation of a standardized communication tool “DC HOME” significantly improved resident performance during the discharge conversation. The use of the “DC HOME” mnemonic also improved patients’ perception regarding a resident physician’s performance during discharge and overall patient satisfaction.

During the pre-intervention phase, in more than 76% of encounters, resident physicians did not ask their patients about obstacles to further care, such as affording medications or transportation to follow-up visits and did not receive education on health and lifestyle modifications, such as quitting tobacco use or improving their diet. Nearly 20% of the time, residents did not provide patients with information regarding newly prescribed medications and did not provide patients with expectations following discharge, including expected course of illness and reasons to return to the ED. These findings may be attributed to a “lack of standardized formal training and evaluation which is not the norm at most emergency medicine training programs as well as a limitation of education priorities based on perception and belief that senior residents are competent.” 13

Implementation of “DC HOME” allows a platform for faculty to assess a resident’s performance and provide feedback after direct observation. Prior studies have shown that the use of direct observation as a formal evaluation of EM residents is valuable to their education, identifies areas requiring improvement, and that the presence of faculty evaluators is not overly intimidating.^15^ The ACGME Milestones project has encouraged the implementation of bedside assessments as a means of ensuring clinical competency.^16^ Implementation of “DC HOME” will provide another tool in resident performance evaluation.

ACGME guidelines for EM residents stress effective patient communication as a core competency.^16^ The ACGME requires formal education in patient handoffs.^17^ The discharge conversation is a perfect opportunity to evaluate this core competency.^16^–^18^ In an effort to reduce errors, communication training and the use of mnemonics to standardize transfer of critical information have been recommended.^19^–^21^ When used in clinical practice, the I-PASS mnemonic (illness severity; patient summary; action list; situation awareness; synthesis) and the I-PASS study group proved that implementation of these recommendations when turnover of patient care between physicians occurs can significantly reduce medical errors and rate of adverse events among hospitalized patients.^22^ The magnitude of the discharge conversation is similar to a patient handoff from physician to physician, with the difference that the responsibility of care is transferred directly to the patient.^23^–^26^ Our study illustrates a significant improvement in the ability of a resident physician to address the most important components necessary to safely transfer care back to the patient. Future studies may look to address “DC HOME” and its impact on recidivism, medical errors, adverse drug events, and malpractice liability.^1^–^4^

Use of the “DC HOME” mnemonic resulted in a statistically significant improvement in overall patient satisfaction (Table 3). Currently healthcare is a competitive business market where healthcare business models, which include value-based incentives, gauge patient satisfaction to improve quality, retain patients and gain market share. In EM, it has been shown that the quality of discharge instructions improves patient satisfaction.^27^–^31^ The time spent in communication between the patient and resident physician increased roughly 66% between our pre-intervention and post-intervention groups. Although there was a statistically significant increase in time spent, it aligns with the results of a similar study by Vashi et al, which had an average amount of time spent during discharge of about four minutes.^10^

In our study we recognize that time management and efficiency is crucial in emergency physicians’ workflow; however, the extra 1 minute and 36 seconds spent in patient/provider discharge communication will align with the patients’ needs and preferences as well as help improve resident competency in important elements of interpersonal skills and performance with communication.^17^

## LIMITATIONS

The main potential limitation to this study was investigator bias, where four authors included in the faculty observers (18 total) cohort performed pre- and post-intervention observations. We recognize that knowledge of the education intervention may have skewed the results toward impact benefit. However, the authors represented only 22% of total observers and performed 46 total observations (20 pre/26 post), which constitutes a small percentage of the sample size. While relevant, this potential bias would have no bearing on the patient perception and patient satisfaction results, which showed improvement.

Another potential limitation is observer bias, whereby resident physicians may have altered their behaviors (consciously or unconsciously) during the discharge process because they were aware that they were being observed. The observer bias may have been more far reaching post-intervention as the residents were aware they were being evaluated on their discharge conversation skills and their ability to implement the “DC Home” mnemonic into their discharge conversation. Future studies can attempt to assess how residents performed with other patients when they were not being observed by faculty.

Satisfactory fulfillment of each discharge component criteria was based on the investigating faculty subjective opinion, limiting the ability to define formal standardized criteria for each of the six components of the discharge discussion, which could had led to confirmation bias, social desirability bias, and acquiescence bias. In an attempt to mitigate these limitations, faculty were provided with example phrases within the questionnaire that if mentioned would constitute a successful acknowledgment of the specific component of the discharge conversation being observed. (Appendix 1). Institution of an inter-rater reliability review and calculation process would have also addressed this limitation; however, this was not included in the study design.

The discharge diagnosis may have been a factor in determining the proficiency of the discharge encounter. For instance, an otherwise healthy young patient with acute pharyngitis may not have received or required a conversation about health and lifestyle modifications and obstacles after discharge; thus, providing a “not applicable” choice option within the questionnaire would have been more inclusive of the variety of discharge diagnoses provided in an ED encounter. This omission may have had an effect on final statistical analysis. While this may be considered a limitation, the binary design of the questionnaire assessed residents’ attempts to make mention of all six components of the discharge mnemonic despite the possibility of a certain component not being applicable. Regardless of the possibility of a component not being applicable, it is our opinion that instruction, repetition, and implementation of a protocolized approach to consistently consider all six components of the “DC HOME” mnemonic will give resident physicians the framework to consistently deliver a comprehensive discharge conversation.

Lastly, the resident’s level of training was not accounted for. All discharge observation evaluations were performed and interpreted independent of level of training. In doing so, data on discharge instructions by level of training was not available, and perhaps those with fewer years of informal discharge training may have been missing more components of the discharge process secondary to less experience. There was a missed opportunity to pair pre- and post-intervention results of each resident along with exploring the possibility of years of training effecting proficiency.

## CONCLUSION

Formal education and the use of a standardized discharge mnemonic “DC HOME” improved emergency medicine resident physicians’ performance at discharge. After implementation, patients perceived residents as more effective communicators at the time of discharge and expressed greater satisfaction with the discharge conversation.

## Supplementary Information



## Figures and Tables

**Table 1:** Attending emergency physicians’ observations of residents’ discharge discussions with patients.

Physician observation PRE		Physician observation POST		P-values
Answer choices	Responses	Answer choices	Responses	
Diagnosis				
Yes	191 (95.5%)	Yes	200 (100%)	0.0036
No	9 (4.5%)	No	0 (0%)	
Care rendered				
Yes	177 (88.5%)	Yes	200 (100%)	<0.0001
No	23 (11.5%)	No	0 (0%)	
Health/lifestyle changes				
Yes	48 (24%)	Yes	193 (96.5%)	<0.0001
No	152 (76%)	No	7 (3.5%)	
Obstacles after discharge				
Yes	45 (22.5%)	Yes	200 (100%)	<0.0001
No	155 (77.5%)	No	0 (0%)	
Medications				
Yes	161 (80.5%)	Yes	200 (100%)	<0.0001
No	39 (19.5%)	No	0 (0%)	
Expectations				
Yes	162 (81%)	Yes	200 (100%)	<0.0001
No	38 (19%)	No	0 (0%)	

Differences between pre- and post-intervention were analyzed with Fisher’s exact test.

P-values ≤0.05 were considered as statistically significant.

**Table 2:** Patient perceptions of resident physicians’ discharge instructions in the emergency department.

Patient responses PRE		Patient responses POST		P-values
Answer choices	Responses	Answer choices	Responses	
Diagnosis				
Yes	189 (94.5%)	Yes	199 (99.5%)	0.0029
No	11 (.5%)	No	1 (0.5%)	
Care rendered				
Yes	198 (99%)	Yes	199 (99.5%)	0.6231
No	2 (1%)	No	1 (0.5%)	
Health/lifestyle changes				
Yes	108 (54%)	Yes	175 (87.5%)	<0.0001
No	92 (46%)	No	25 (12.5%)	
Obstacles after discharge				
Yes	95 (47.5%)	Yes	178 (89%)	<0.0001
No	105 (52.5%)	No	22 (11%)	
Medications				
Yes	157 (78.5%)	Yes	192 (96%)	<0.0001
No	43 (21.5%)	No	8 (4%)	
Expectations				
Yes	174 (87%)	Yes	196 (98%)	<0.0001
No	26 (13%)	No	4 (2%)	

Differences between pre- and post-intervention were analyzed with Fisher’s exact test.

P-values ≤0.05 were considered as statistically significant.

**Table 3:** Patient satisfaction scores before and after introduction of a standardized communication tool for discharge conversations.

Satisfaction scores	
Answer choices	Responses
Pre-intervention	
0 = not satisfied	2 (1%)
3 = somewhat satisfied	27 (13.5%)
5 = very satisfied	171 (85.5%)
Post-intervention	
0 = not satisfied	0 (9%)
3 = somewhat satisfied	4 (2%)
5 = very satisfied	196 (98%)
Fisher 2×3 table:	0.005

Differences between pre and post-intervention were analyzed with Fisher’s 2 × 3 variant test.

P-values ≤0.05 were considered as statistically significant.

**Table 4:** Length of time spent by resident physicians during the discharge conversations with patients.

Pre-intervention (time in seconds)	162
Post-intervention (time in seconds)	244
Unpaired t-test	<0.001

Differences between pre and post-intervention were analyzed using the unpaired t-test.

P-values ≤ 0.5 were considered as statistically significant.
