# Supplementary material for: Does a Standardized Discharge Communication Tool Improve Resident Performance and Overall Patient Satisfaction?
Source: West J Emerg Med. 2020 Nov 20;22(1):52–9. doi: 10.5811/westjem.2020.9.48604 (PMC7806327; doi:10.5811/westjem.2020.9.48604)
Supplement: Supplementary file 1 [file wjem-22-52-s001.docx]

**APPENDIX 1**

Discharge observation evaluation. The 6 components of the discharge discussion that were evaluated are listed below with a binary response (“Yes” or “No”).

|  | Yes | No |
| --- | --- | --- |
| Did the resident physician explain the patient’s diagnosis? |  |  |
| Did the resident physician explain the diagnostic tests performed in the Emergency Department, medications administered, and a summary of the results? |  |  |
| Did the resident physician recommend healthy lifestyle changes such as quitting tobacco, limiting alcohol, modifying their diet, being physically active every day, and/or reducing stress? |  |  |
| Did the resident physician address the need for social work? (i.e. Can the patient afford and fill medications? Does the patient have transportation? Does the patient have good mobility? Does the patient live alone? Is the patient able to perform activities of daily living? Does the patient have assistance at home? Does the patient have a Primary Care Provider?) |  |  |
| Did the resident physician provide instructions on how to take newly prescribed medications or to stop taking any current medications? |  |  |
| Did the resident physician provide information about when symptoms should improve, when and whom to follow up with and reasons to return to the EmergencyDepartment? |  |  |
